# Patient satisfaction and outcomes of crisis resolution home treatment for the management of acute psychiatric crises: a study during the COVID-19 pandemic in Madrid

**DOI:** 10.3389/fpsyt.2023.1197833

**Published:** 2023-09-05

**Authors:** Irene Moreno-Alonso, Manuel Nieves-Carnicer, Alexandra Noguero-Alegre, Miguel Angel Alvarez-Mon, Alberto Rodriguez-Quiroga, Juan F. Dorado, Fernando Mora, Javier Quintero

**Affiliations:** ^1^Department of Psychiatry and Mental Health, Hospital Universitario Infanta Leonor, Madrid, Spain; ^2^Department of Medicine and Medical Specialities, University of Alcala, Alcala de Henares, Madrid, Spain; ^3^Ramón y Cajal Institute of Sanitary Research (IRYCIS), Madrid, Spain; ^4^PeRTICA Análisis Estadísticos, Madrid, Spain; ^5^Department of Legal and Psychiatry, Complutense University, Madrid, Spain

**Keywords:** crisis resolution home treatment, COVID-19 pandemic, acute psychiatric crises, inpatient treatment, caregiver burden

## Abstract

**Background:**

Crisis Resolution Home Treatment (CRHT) seem to offer comparable results to the traditional hospitalization model, at a lower cost and offering greater flexibility and scope. However, in Madrid, its implementation in Mental Health did not occur until the midst of the COVID-19 pandemic. In this work we analysed the effectiveness of a mental health CRHT unit promoted during the COVID-19 pandemic, as well as the degree of satisfaction of patients and their families.

**Methods:**

90 patients were treated by the CRHT unit in the period between October 2020 and June 2022. All patients met the inclusion criteria: (1) Acute psychopathological decompensation in patients suffering from psychotic disorders, major affective disorder, obsessive compulsive disorder, personality disorder and other severe mental disorders causing functional disability, according to ICD-10 diagnostic criteria; (2) Ages between 18–90 years old; (3) Living in the urban area of Vallecas, Madrid; and (4) Counting with sufficient social and family support. The effectiveness of the intervention was evaluated with the SF-36 health questionnaire, the caregiver burden with the Zarit questionnaire, and patient satisfaction with a survey specifically designed for this work.

**Results:**

55 (61.1%) patients completed the SF-36 at baseline and at the end of hospitalization. Statistically significant improvements were observed in the 8 dimensions of the SF-36 (*p* < 0.05). However, CRHT did not achieve a statistically significant decrease in caregiver burden. Regarding the satisfaction of the patients with the attention and care received, an average score of 47.72/50 was obtained.

**Conclusion:**

The Crisis Resolution Home Treatment intervention resulted in significant improvement in patients’ quality of life with high satisfaction scores. However, it did not effectively reduce caregiver burden. Future research should focus on randomized controlled trials with long-term follow-up to assess the effectiveness of CRHT compared to traditional hospitalization and utilize specific assessment scales for different mental disorders.

## Introduction

The emergence of Crisis Resolution Home Treatment (CRHT) units in medicine, which provide care teams similar to hospital care but located in the patient’s home, began in the mid-20th century in the United States and spread throughout Europe in the second half of the century (1). While these units vary in characteristics and resources, they are well-integrated care models documented in the literature, particularly from the United States, England, Australia, Italy, and Spain (2, 3). In the field of psychiatry, this model of care is primarily found in Anglo-Saxon countries such as the United States, England, and Australia, with England implementing CRHT nationwide since 2000 (4).

Although the scientific evidence is still limited, the popularity of CRHT could be attributed to its comparable clinical outcomes to traditional hospitalization, lower costs, and increased flexibility and scope for healthcare services (5, 6). A recent systematic review evaluating CRHT studies across multiple countries suggested that it may be a promising alternative to hospital admission, although further research is needed to understand its potential drawbacks or disadvantages compared to traditional hospitalization (7). In the realm of mental health, CRHT may also contribute to reducing stigma (8–10). The COVID-19 pandemic highlighted the importance of CRHT as hospitals faced bed, staff, and supply shortages. In response, various medical specialties in Spain established home hospitalization units to prevent readmissions and deliver intensive treatments amidst hospital capacity constraints (11, 12).

Following the pandemic, there has been a gradual increase in the demand for psychiatric and mental health care in hospitals (13). This demand has been particularly high in the urban area of Vallecas, Madrid, with significant pressure on the Brief Psychiatric Hospitalization Unit (BPHU) from the emergency department and a consistently high occupancy rate of around 90%. In response to this situation, the Mental Health Crisis Resolution Home Treatment (CRHT) unit was established in this area.

The objectives of our study are as follows: (1) to describe the demographic characteristics, referral criteria, and pathology of the patients treated at the Mental Health CRHT; (2) to analyze the effectiveness of the program in terms of overall patient improvement; (3) to assess patient satisfaction with the Mental Health CRHT through a satisfaction survey; (4) to identify which patients benefit the most from CRHT and which patients are most satisfied; and (5) to evaluate the potential benefits in terms of caregiver burden.

## Methods

### Patients and study protocol

The Vallecas Mental Health Crisis Resolution Home Treatment (CRHT) Unit operates with a team consisting of 3 psychiatrists and 3 mental health nurses, available from 8 a.m. to 3 p.m. on weekdays. Outside of these hours, patients and their families have access to a 24-h service number for emergency situations, providing immediate telephonic assistance and on-call staff for in-person assessments when required. The workload is divided into teams, with each team comprising one psychiatrist and one nurse. Home visits are conducted by one or two teams, performing comprehensive assessments, evaluations, vital sign measurements, and diagnostic tests as needed. The remaining team at the hospital collaborates with other medical specialties, coordinates further diagnostic procedures, and prepares reports or evaluations for admissions.

**Figure 1:**
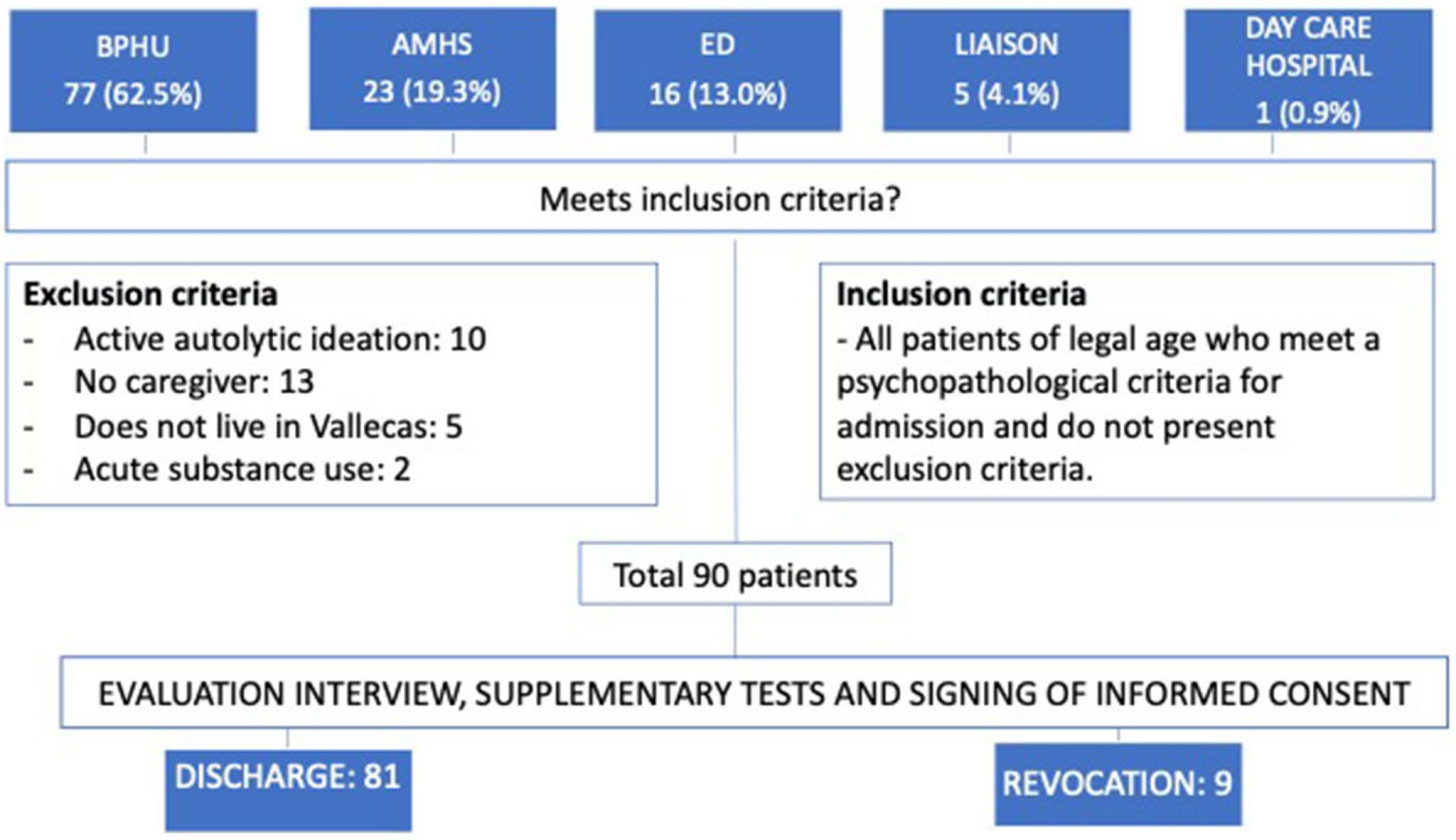
Flowchart of referrals to CRHT.

Patients access the CRHT Unit through referrals from the Brief Psychiatric Hospitalization Unit (BPHU), Adult Mental Health Services (AMHS), and the Emergency Department (ED). The case manager evaluates the patients and refers them to the CRHT Unit based on specific criteria, including psychopathological decompensation in severe mental disorders causing functional disability, age between 18–90 years, residence in the urban area of Vallecas, Madrid, and having sufficient social and family support. Patients at risk of suicide or actively using substances are excluded. Informed consent is obtained from patients before receiving care at their homes, explaining the program, objectives, and rules (see Figure 1).

A total of 122 patients were evaluated between October 2020 and June 2022, with 32 patients being excluded. Although most patients met the inclusion criteria, there were instances of admission revocations due to reasons such as drug overdose, suicide, hospital readmission, and substance use.

### Assessment instruments and variables considered

During the first interview, sociodemographic data was collected (Table 1), and patients were assessed using the following measurement instruments: (1) SF-36 Health Questionnaire (Table 2), (2) Patient satisfaction survey, and (3) Zarit Scale of Caregiver Burden (Table 3). The sociodemographic data included age, sex, nationality, marital status, cohabitation, level of education, and professional activity. The SF-36 questionnaire is a validated scale consisting of 36 questions that assess various dimensions of health status (14, 15). The Zarit Scale of Caregiver Burden is a scale with 22 questions that measure the burden experienced by caregivers (16, 17). The satisfaction survey comprises 20 multiple-choice questions addressing different aspects of the treatment experience, such as information management, explanation of the therapeutic plan, medication effects, and program functioning (Supplementary material). It also covers satisfaction with the professionals’ treatment, incident handling, coordination among professionals, admission process, and willingness to choose this therapeutic modality again if needed.

**Table 1:** Sociodemographic characteristics of the sample.

Age, mean	47.29
Sex, *n* (%)	
Man	32 (35.56%)
Female	58 (64.44%)
Mean stay	23.59
Referral resource	
BPHU	55
ED	14
AMHS	17
Others (Psychiatric Day Hospital and Liaison and Interconsultation Psychiatry)	4
Preadmission working status	
Active	20
Unemployed or homemaker	33
Student	5
Pensioner	32
Diagnoses	
Other disorders	4 (4.44%)
Psychotic disorders	43 (47.78%)
Bipolar disorders	12 (13.33%)
Depressive disorders	12 (13.33%)
Anxiety disorders	4 (4.44%)
Personality disorders	15 (16.67%)

**Table 2:** Statistical results of the SF-36 scale comparing pre and post treatment.

	Pre mean	Post mean	MV	SD MV	*p*
Physical function	66.36	75.64	9.27	17.65	<0.0001
Physical role	12.73	32.73	20.00	39.21	0.0002
Emotional role	21.82	43.03	21.21	41.75	0.0001
Vitality	34.91	49.82	14.91	22.66	<0.0001
Mental health	39.35	52.82	13.47	27.36	0.0003
Social function	33.41	50.91	17.50	32.42	0.0002
Physical pain	51.90	66.73	14.83	37.83	0.0053
General health	43.00	51.27	8.27	18.71	0.0018

MV: mean of the variance between the pre and post treatment results. SD, standard deviation. *p*, *p* value corresponding to MV.

**Table 3:** Statistical results of the Zarit scale of Caregiver Burden comparing pre and post treatment.

	*N*	Pre mean	Post mean	MV	SD	*p*
ZARIT	54	51.91	47.35	−4.56	15.62	0.0605
ZARIT SMD	34	–	–	−0.74	7.60	0.0176*
ZARIT MMD	20	–	–	−11.1	22.59	

*The resulting p corresponds to the statistical analysis between the variation of SMD and MMD. MV: mean of the variance between the pre and post treatment results. SD, standard deviation. *p*, *p* value corresponding to MV. ZARIT SMD, Zarit severe mental disorder; ZARIT MMD, Zarit mild mental disorder.

### Ethical considerations

The study was approved by the Clinical Research Ethics Committee of the University Hospital Infanta Leonor (reference: 044–23).

### Statistics

The pre-post treatment changes in the different domains of the SF-36 scale were evaluated using the unpaired T-Student test, and in cases where the assumptions of the test were not met, the Sign test was applied. For qualitative variables, such as the Zarit test, changes over time were measured using the Bowker Symmetry Test. Group comparisons were conducted using Student’s *T*-test, and in cases where the assumptions of the test were not met, the Mann–Whitney U-test was used. When there were more than two groups, an ANOVA model or, in the case of non-homogeneous variances, the non-parametric Kruskal-Wallis test was employed. The significance level was set at *p* < 0.05. The data analysis was performed using SAS 9.4 software by SAS Institute Inc., Cary, NC, United States.

## Results

### Demographic patient characteristics

The patients in the study had an average age of 47.29, with a majority of women (64.44%), and their average duration of stay in the CRHT was 23.59 days (Table 1). The Brief Psychiatric Hospitalization Unit (BPHU) was the primary referral source. In terms of employment, two main groups stood out: unemployed or homemakers and pensioners.

The most common diagnoses among these patients were psychotic disorders (47.89%), followed by personality disorders, bipolar disorder, and depressive disorders (Table 1). To present the data, the diagnoses were grouped into six categories: psychotic disorders, bipolar disorders, depressive disorders, anxiety disorders, personality disorders, and other disorders. For statistical analyses, the pathologies were further grouped into two categories: severe mental disorder (SMD) consisting of psychotic disorders and bipolar disorders, and mild mental disorder (MMD) consisting of other disorders, depressive disorders, anxiety disorders, and personality disorders.

### The intervention carried out in the crisis resolution home treatment unit significantly improves the quality of life of patients

Out of the total patients, 55 (61.1%) completed the SF-36 questionnaire at the beginning and end of their hospitalization. Significant improvements were observed in all eight dimensions of the SF-36. On average, patients showed improvements of 9.27 points in physical function, 20 points in physical role, 21.21 points in emotional role, 14.91 points in vitality, 13.47 points in mental health, 17.5 points in social function, 14.83 points in bodily pain, and 8.27 points in general health (Table 2).

There were no significant differences between men and women in terms of changes in physical function, physical role, emotional role, mental health, social function, pain, or general health (Data not shown). However, significant differences were observed in vitality, with women benefiting more from the intervention. Significant differences were also found in the variation of physical function between different age groups, with those over 48 years of age benefiting the most (Data not shown). No significant differences were observed for the other dimensions (physical role, emotional role, vitality, mental health, social function, pain, and general health).

There were no statistically significant differences in the SF-36 scores (or any of its dimensions) based on the patients’ specific mental health diagnoses. This means that patients with severe mental disorders (SMD) benefited from the intervention in the same way as patients with milder mental disorders.

### The patients showed a very high level of satisfaction with the intervention

The average satisfaction score obtained was 47.72 out of 50, indicating a high level of satisfaction. No statistically significant differences were found in the overall satisfaction level based on the patients’ sex, age, or diagnosis (Table 3).

Regarding caregiver burden, 54 caregivers responded before and after the intervention. There were no statistically significant differences observed between pre and post hospitalization in terms of caregiver burden. In other words, the home hospitalization did not lead to a significant reduction in caregiver burden overall. No significant differences were found in the scores between pre and post intervention based on the type of relationship between caregivers and patients (parent/child, partner, or other), nor based on the caregivers’ sex or age. However, significant differences were observed between caregivers of patients with severe mental illness and caregivers of patients with milder mental disorders. Caregivers of patients with milder pathology showed a significantly greater improvement in burden compared to caregivers of patients with severe mental disorders.

## Discussion

In this study, we examined the impact of the Crisis Resolution Home Treatment (CRHT) intervention provided by the Infanta Leonor University Hospital in Madrid. We assessed the effectiveness of the intervention using the SF-36 questionnaire and found statistically significant improvements in all dimensions, regardless of pathology, intervention duration, or demographic variables. The greatest improvements were observed in the Emotional Role and Physical Role dimensions, followed by Social Function, Vitality, Pain, and Mental Health. Caregiver burden was measured using the Zarit scale, and although no statistically significant differences were found, there was a clear trend towards reduced burden. However, when grouping pathologies into Severe Mental Disorders (SMD) and Mild Mental Disorders (MMD), statistically significant differences were obtained, indicating a greater reduction in burden for caregivers of patients with MMD compared to those with SMD. Patient satisfaction with the care received was also assessed, and the average score obtained was 47.72 out of 50.

Comparing our findings with data from similar units in other European countries, we did not find notable differences in terms of demographic and clinical characteristics of our sample (18–21). The only notable difference was a higher number of admissions with a primary diagnosis of psychotic disorders compared to studies in other European countries (19–21). However, this aligns with the incidence of psychotic disorders in the Spanish population (18). Additionally, we observed a higher incidence of personality disorders compared to previous studies, of which could be influenced by the COVID-19 pandemic and its impact on mental health (22). Studies have reported worsened mental health symptoms and difficulties accessing healthcare during the pandemic, particularly for individuals with personality disorders (23, 24). The CRHT intervention provided specialized care to patients during a time when access to hospitals was limited. Future studies could investigate whether the diagnostic distribution returns to previous patterns once the pandemic subsides.

When evaluating the changes observed in the SF-36 questionnaire, it is important to consider certain aspects. Firstly, there was a significant loss of data, as only 55 out of the 90 patients who completed the scale before admission also completed it on discharge, resulting in a reduced sample size. Despite this, statistical significance was achieved in the perceived improvement across all subscales, regardless of the diagnosis. This indicates that the quality of life of patients substantially improves after receiving intervention from the Mental Health CRHT unit. However, we were unable to find other studies directly comparing the results of the SF-36 in CRHT patients in psychiatry, so we do not have a direct benchmark for the effectiveness of our intervention. Nevertheless, there are studies that correlate the results and variations of the SF-36 with specific scales of psychiatric symptoms such as the BSI, Hamilton-D, and HoNOS, as well as the clinical status of the patient (25–27).

On the other hand, the HoNOS scale has been used to assess the effectiveness of the home treatment model (18, 19, 21). In fact, one study compared the clinical outcomes of home treatment and conventional hospitalization using this scale and found comparable results, although hospitalization at home showed longer mean stays (21). Therefore, the observed improvement in the SF-36 scores is promising in terms of the intervention’s effect on patients, but future studies using other validated clinical scales are needed to compare the results with similar units and traditional hospitalization.

The results obtained from the Zarit scale prior to the intervention indicate the high burden experienced by caregivers of patients with mental illness, particularly those with psychotic disorders requiring hospitalization, as in our case. These results align with previous studies (28, 29). However, it was hypothesized that the home intervention, which includes caregivers, could help reduce this burden. Although the results support this hypothesis, the reduction in burden did not reach statistical significance, albeit showing a slight trend when considering the entire sample. It should be noted that data after the intervention were lost again, and the variation could only be analyzed in 56 caregivers, significantly reducing the sample size. With a larger number of data, statistical significance might have been achieved. However, the average improvement in the score was modest, leaving the average burden levels still very high. Only when stratifying the analysis between SMD and other diagnoses did a clinically and statistically significant difference emerge, favoring the latter group. While no specific previous studies have been found on the effect of CRHT on caregiver burden, this result can be explained from different perspectives. Firstly, it is possible that the temporal distance between our measures (with a mean stay of around 23 days) is insufficient to assess the effect of the intervention on the most chronic and severe disorders such as SMD (30). Secondly, studies have found that the admission of another profile of chronically ill patients to an institution significantly reduces caregiver burden (31). Thus, it is plausible that in the context of home admission and the need to continue caring for the patient during the crisis, caregiver burden persists despite specific support interventions.

The perception of satisfaction has yielded highly favorable results, indicating a high level of acceptance among users of this care modality. However, it is important to consider that the satisfaction survey is conducted in person, on paper, by the professionals directly responsible for the patient. This introduces a significant social desirability bias that may affect the realism of these excellent results, as demonstrated in previous studies (32–35).

The present study has several important limitations. Firstly, the sample size is small and limited to a single hospital and a specific area with unique sociodemographic characteristics, which restricts the generalizability of the findings. Furthermore, the narrow time frame for analyzing clinical changes prevents the assessment of medium-and long-term effects of the intervention. Additionally, due to differences in unit characteristics and scales used in other studies, it is challenging to directly compare the results obtained. Moreover, a comparison with the standard model of hospitalization was not conducted. Finally, it is worth noting that self-applied scales were used as assessment instruments, and the reliability of the results could be enhanced by employing hetero-applied scales and questionnaires administered by medical staff.

## Conclusion

The results of the study indicate a significant improvement in the quality of life of patients and overall health outcomes following the CRHT intervention. The high level of patient satisfaction also reflects the positive impact of the intervention. However, it was not possible to reduce caregiver burden in the short term, particularly for caregivers of patients with severe mental disorders. Future research should focus on conducting randomized controlled trials with long-term follow-up to assess the effectiveness of this therapeutic approach compared to traditional hospitalization. Additionally, the use of specific assessment scales tailored to each mental disorder should be considered for more accurate evaluation.

## Data availability statement

The raw data supporting the conclusions of this article will be made available by the authors, without undue reservation.

## Ethics statement

The studies involving human participants were reviewed and approved by Hospital Infanta Leonor Ethics Committee. The patients/participants provided their written informed consent to participate in this study. The studies were conducted in accordance with the local legislation and institutional requirements.

## Author contributions

All authors listed have made a substantial, direct, and intellectual contribution to the work and approved it for publication. The first five authors conducted the fieldwork, the sixth author performed the statistical analysis, and the seventh and eighth authors were responsible for study design. Additionally, the first and second authors handled the manuscript writing, and all authors contributed by critically reviewing it.

## Conflict of interest

The authors declare that the research was conducted in the absence of any commercial or financial relationships that could be construed as a potential conflict of interest.

## Publisher’s note

All claims expressed in this article are solely those of the authors and do not necessarily represent those of their affiliated organizations, or those of the publisher, the editors and the reviewers. Any product that may be evaluated in this article, or claim that may be made by its manufacturer, is not guaranteed or endorsed by the publisher.

## Supplementary material

Supplementary Datasheet 1 and 2 Satisfaction survey.
