# Supplementary material for: Patient satisfaction and outcomes of crisis resolution home treatment for the management of acute psychiatric crises: a study during the COVID-19 pandemic in Madrid
Source: Front Psychiatry. 2023 Sep 5;14:1197833. doi: 10.3389/fpsyt.2023.1197833 (PMC10507704; doi:10.3389/fpsyt.2023.1197833)

**ENCUESTA DE SATISFACCIÓN**

Los miembros de la unidad de hospitalización a domicilio para personas con enfermedad mental del Hospital Universitario Infanta Leonor que le hemos atendido estamos interesados en conocer su opinión acerca de la calidad del servicio que se le ha ofrecido. Por este motivo nos permitimos dirigirnos a usted con el propósito de solicitar su colaboración, con la seguridad de que su opinión y ayuda serán de gran utilidad para que podamos hacerlo mejor.

Por supuesto, su colaboración es totalmente voluntaria y completamente anónima. Todo lo que escriba será tratado de forma absolutamente confidencial. Nos permitimos sugerirle que cumplimente cuanto antes el cuestionario, para que no olvide hacerlo. Le agradecemos muy sinceramente su interés y su atención para con nuestro trabajo. Muchas gracias.

Edad________

Profesión______________________________________________

| Sexo: |  | Hombre |  | Mujer |
| --- | --- | --- | --- | --- |

Marque aquella opción que mejor refleje su opinión:

1. ¿Entendió las explicaciones que se dieron en el momento de decidir su ingreso hospitalario?

☺ 😐 ☹

🞏 🞏 🞏

1. Cuando ingresó en el programa, ¿le entregaron un folleto informativo?

|  | SI |  | NO |
| --- | --- | --- | --- |

1. Valore el trato recibido por el médico

☺ 😐 ☹

🞏 🞏 🞏

1. Valore el trato recibido por la enfermera

☺ 😐 ☹

🞏 🞏 🞏

1. La información que le dio el médico la calificaría como

☺ 😐 ☹

🞏 🞏 🞏

1. La información que le dio la enfermera la calificaría como

☺ 😐 ☹

🞏 🞏 🞏

1. ¿Le pareció adecuado el plan de tratamiento en el momento del ingreso domiciliario?

☺ 😐 ☹

🞏 🞏 🞏

1. ¿Fue informado/a sobre el modo de tomar la medicación y sus efectos secundarios?

☺ 😐 ☹

🞏 🞏 🞏

1. A veces para tratar una misma enfermedad hay diferentes alternativas o tratamientos ¿le pidió su médico opinión sobre las alternativas disponibles?

|  | Sí |  | NO |
| --- | --- | --- | --- |

1. ¿Se solucionaron adecuadamente las situaciones difíciles que se presentaron?

☺ 😐 ☹

🞏 🞏 🞏

1. ¿Tuvo necesidad durante su ingreso domiciliario de acudir al servicio de urgencias?

|  | Sí |  | NO |  |  | NO |
| --- | --- | --- | --- | --- | --- | --- |

1. ¿Cómo valoraría la coordinación de los distintos profesionales que le han atendido?

☺ 😐 ☹

🞏 🞏 🞏

1. En el momento del alta, ¿los médicos o las enfermeras le dieron instrucciones precisas sobre el tratamiento a seguir?

|  | SI |  | NO |
| --- | --- | --- | --- |

1. ¿Entendió las instrucciones que le dieron?

☺ 😐 ☹

🞏 🞏 🞏

1. Al alta ¿le han dado cita para su Centro de Salud Mental?

|  | SI |  | NO |
| --- | --- | --- | --- |

1. En general ¿Cómo valoraría la asistencia que recibió durante el ingreso domiciliario?

☺ 😐 ☹

🞏 🞏 🞏

1. En caso de volver a necesitar ingreso, ¿volvería a escoger este tipo de ingreso en lugar del ingreso hospitalario?

|  | SI |  | NO |
| --- | --- | --- | --- |

1. ¿Cómo se encuentra de su dolencia?

☺ 😐 ☹

🞏 🞏 🞏

1. ¿Se ha resuelto satisfactoriamente la situación que motivó el ingreso domiciliario?

|  | SI |  | NO |
| --- | --- | --- | --- |

1. Ha terminado de cumplimentar el cuestionario. Gracias por su interés y colaboración. Si cree preciso incluir alguna consideración o comentario más, puede hacerlo a continuación:

|  |
| --- |
|  |
|  |
|  |
|  |
|  |
|  |
|  |
|  |
|  |


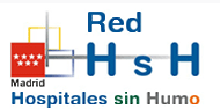

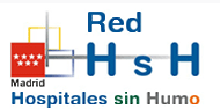

Supplement: Supplementary Datasheet 1 and 2 — Satisfaction survey. [file Data_Sheet_1.docx]
