# Supplementary material for: Patient satisfaction and outcomes of crisis resolution home treatment for the management of acute psychiatric crises: a study during the COVID-19 pandemic in Madrid
Source: Front Psychiatry. 2023 Sep 5;14:1197833. doi: 10.3389/fpsyt.2023.1197833 (PMC10507704; doi:10.3389/fpsyt.2023.1197833)
Supplement: Supplementary file 2 [file Data_Sheet_2.docx]

SATISFACTION SURVEY

The members of the Home Hospitalization Unit for individuals with mental illness at the Infanta Leonor University Hospital who have provided care for you are interested in knowing your opinion about the quality of the service provided to you. For this reason, we kindly request your collaboration, with the assurance that your opinion and assistance will be of great help for us to improve.

Your participation is completely voluntary and anonymous. Everything you write will be treated with absolute confidentiality. We suggest that you complete the questionnaire as soon as possible, so you don't forget to do it. We sincerely appreciate your interest and attention to our work. Thank you very much.

Age: ________ Profession: ______________________________________________

| Sex: |  | Man |  | Woman |
| --- | --- | --- | --- | --- |

Please select the option that best reflects your opinion:

1. Did you understand the explanations given at the time of deciding your home hospitalization?

☺ 😐 ☹

🞏 🞏 🞏

1. When you entered the program, were you provided with an informational booklet?

|  | YES |  | NO |
| --- | --- | --- | --- |

1. Rate the treatment received from the doctor.

☺ 😐 ☹

🞏 🞏 🞏

1. Rate the treatment received from the nurse.

☺ 😐 ☹

🞏 🞏 🞏

1. How would you rate the information provided by the doctor?

☺ 😐 ☹

🞏 🞏 🞏

1. How would you rate the information provided by the nurse?

☺ 😐 ☹

🞏 🞏 🞏

1. Did you find the treatment plan appropriate at the time of home hospitalization?

☺ 😐 ☹

🞏 🞏 🞏

1. Were you informed about how to take the medication and its side effects?

☺ 😐 ☹

🞏 🞏 🞏

1. Sometimes, for the treatment of the same condition, there are different alternatives or treatments. Did your doctor ask for your opinion on the available alternatives?

|  | YES |  | NO |
| --- | --- | --- | --- |

1. Were difficult situations adequately resolved?

☺ 😐 ☹

🞏 🞏 🞏

1. Did you need to go to the emergency department during your home hospitalization?

|  | YES |  | NO |
| --- | --- | --- | --- |

1. How would you rate the coordination among the different professionals who attended to you?

☺ 😐 ☹

🞏 🞏 🞏

1. At discharge, did the doctors or nurses provide you with precise instructions about the follow-up treatment?

|  | YES |  | NO |
| --- | --- | --- | --- |

1. Did you understand the instructions they gave you?

☺ 😐 ☹

🞏 🞏 🞏

1. Were you given an appointment for your Mental Health Center at the time of discharge?

|  | YES |  | NO |
| --- | --- | --- | --- |

1. Overall, how would you rate the care received during your home hospitalization?

☺ 😐 ☹

🞏 🞏 🞏

1. If you needed hospitalization again, would you choose this type of home hospitalization instead of traditional hospitalization?

|  | YES |  | NO |
| --- | --- | --- | --- |

1. How is your condition now?

☺ 😐 ☹

🞏 🞏 🞏

1. Has the situation that led to your home hospitalization been satisfactorily resolved?

|  | YES |  | NO |
| --- | --- | --- | --- |

1. You have completed the questionnaire. Thank you for your interest and cooperation. If you feel it is necessary to include any additional considerations or comments, you can do so below:

|  |
| --- |
|  |
|  |
|  |
|  |
|  |
|  |
|  |
|  |
|  |
